# Fracture of Femur in an Infant

**Published:** 1872-08

**Authors:** A. A. Lyon

**Affiliations:** Columbus, Miss.


### FRACTURE OF FEMUR IN AN INFANT.

BY A. A. LYON, M. D., COLUMBUS, MISS.

On the 11th of March last, I was called to see Sarah W—, a child fifteen months of age, who was reported to have received an injury of the thigh. Upon examination, a distinct and complete fracture of the thigh-bone, about an inch above the junction of the middle with the lower third was clearly discovered. It appeared that the nurse, with the child in her arms, had fallen, and in the fall had produced this injury.

The extreme youth of the patient, the comparative rarity of such cases, and the want of specific data for the treatment of such, led us to apprehend possible trouble, and unsatisfactory results.

Of course, in a child of such tender years, requiring to be frequently moved in changing its clothes, &c., it was altogether impracticable to resort to a fixed apparatus, including permanent extension and counter-extension, as in the case of adults or older children. In addition to this, another difficulty was presented in the contamination of the dressings by the urine and other discharges of the patient—a result that no possible care could entirely prevent, and which we feared might aid in producing painful, and may be, ulcerous excoriation.

Under these circumstances, we decided upon the following plan of treatment, as possibly promising the best results:

Two pieces of binders' board, somewhat thinner than that usually sold for surgical purposes, were cut so as accurately to fit the parts—the inner-splint to extend from the groin to the knee; the outer, from the trochanter to a point midway between the knee and the ankle. The limb was then extended and brought within the proper plane, the fracture adjusted as accurately as possible, and a roller bandage applied from the toes to the groin. The splints, previously soaked in warm water, were then applied and fitted so as to enclose the thigh, as it were, in a tube. Over this two other rollers were applied, and secured in the usual way, an ordinary stocking slipped on and the top tacked to the bandages. This served the better to keep the dressings in their place, and to prevent their soiling.

The little patient was then transferred to a portable bed, which in this instance consisted of the lid of an old trunk, with a pillow placed upon it. The object of this contrivance was for the convenience of changing positions, as circumstances might require in nursing, dressing, &c. To the bottom of this bed, immediately under the limb, and parallel with its plane, was nailed a stout strip of wood, extending ten or twelve inches beyond the foot; to the end of this, and at right angles with it, was attached an upright post of the same dimensions; in the top of which was fixed a pulley. A little cloth gaiter, very like a shoe without a sole, was fitted upon the foot; two tapes, terminating in one—or rather in a cord, were secured to its lower margins. This cord was passed over the pulley, and a bag containing one and a half or two pounds of shot was attached to the end by a hook, so as to be easily removed when necessary. The patient was ordered to be kept as quiet as possible, and great care to be observed in protecting the dressings with the rubber cloth.

The pulley-weight apparatus will be recognized as substantially that of Dr. Gurdon Buck, for producing extension in fractures of the thigh. In this case, however, it was applied, not so much for the purpose of extension as to fix the limb of the little patient, without entirely abridging all motion. In the case of an active, vigorous babe of that age, it was not deemed desirable to confine the parts *absolutely*, neither, indeed—for reasons before indicated was it practicable.

No attempt whatever at counter-extension was made—not even the extension of the foot of the bed, as prescribed by Dr. Buck in the use of his appliance. Indeed, we relied very little upon extension even—not because we did not think it desirable, but because deemed it impracticable. If, however, the ingenuity of other surgeons can suggest in addition to our treatment, a convenient and efficient extension and counter-extension, we would recommend it as possibly promising the most satisfactory cure.

In the treatment of this case, we were apprehensive, as before intimated, of two untoward results. First, deformity in consequence of the frequent and necessary change in the posture of the limb. Second, irritation and excoriation of the soft tissues, from the absorption by the dressings of the discharges. Fortunately for the conduct of our case, as regards the first objection, our patient was remarkably good-natured, and lay very quietly during the whole period of its confinement, giving us no trouble whatever by her restlessness. The dressings were, however, very much saturated and contaminated by the urine, &c. and through a want of proper attention, more perhaps than was altogether necessary. As a consequence, conjoined with the chafing of the upper edges of the splints and bandages, considerable excoriation was produced, involving the entire circumference of the thigh; and we were a little uneasy as to possible results underneath the dressings. As the child, however, continued fat, healthy and quiet, and seemed in all other respects to be doing well, I determined not to endanger the healing fracture by a change, but continued the dressings as first applied for three weeks exactly, preferring to take the risk of injury to the soft parts. On the *twenty-first* day I removed the splints, a little sooner than I would have preferred to do, on account of the sores which seemed to be increasing in gravity day by day.

Upon stripping the limb, I found in addition to the excoriations above referred to, another more serious in the flexure of the knee. This latter seemed to be caused by a strip of roller that had slipped so as to leave exposed a part of the skin. A more careful application would have prevented this. The fracture was found firmly knit, and without any deformity, unless it be a very little shortening—so slight as scarcely to be appreciable. I think it likely the patient might have been put upon her feet immediately, but deemed it best, under the circumstances, to confine her a few days longer. A child, of course, could have no knowledge of its condition, and could exercise no discretion in the management of itself, and hence should not be trusted till the cure is absolutely complete. I ordered it therefore to be kept in bed a week or ten days longer, and applied loosely to the side of the limb a straight splint, merely to keep it quiet and in place.

The excoriations were treated with dressings of simple cerate, and healed perfectly in a few days.

At this juncture I was called away, and did not see my patient again for two weeks. When I returned I found it running around in the yard, apparently as well as it ever did. The mother informed me that they allowed it to get up and walk four or five days after I left, which was less than four weeks from the time of the injury.

In standing the patient up, and comparing the two limbs, certainly no unsurgical eye could have determined which had been the injured one. I feel sure that the slight shortening will entirely disappear with the growth of the child. The result may therefore be considered as eminently satisfactory.

I would remark, that I regard the binders' board, prepared and applied as I have described, as affording perhaps the best possible splint that could be used in such cases. It possesses two most desirable properties: first, that of perfect coaptation to the parts, and next, that of immovable firmness (after it dries). Both of these will be recognized as essential qualities in a splint used in treating fractures of restless children.

And still further, in order to obviate all danger or inversion or eversion of the foot, it should be guarded by a box completly covering it in, or a wire made so as to prevent all contact with the bedding or other external agents.

